# Controlling the Crack Propagation Path of the Veil Interleaved Composite by Fusion-Bonded Dots

**DOI:** 10.3390/polym11081260

**Published:** 2019-07-30

**Authors:** Guangchang Chen, Jindong Zhang, Gang Liu, Puhui Chen, Miaocai Guo

**Affiliations:** 1 State Key Laboratory of Mechanics and Control of Mechanical Structures, Nanjing University of Aeronautics and Astronautics, Nanjing 210016, China; 2 National Key Laboratory of Advanced Composites, AVIC Composite Technology Center, Beijing 101300, China; 3 Center for Advanced Low-Dimension Materials, Donghua University, Shanghai 201600, China

**Keywords:** toughness, structure, composite, fracture, morphology

## Abstract

This study investigated the effect of the fusion-bonded dots of veil interleaves on the crack propagation path of the interlaminar fracture of continuous carbon fiber reinforced epoxy resin. Two thin fiber layers (i.e., nylon veil (NV) with fusion-bonded dots and Kevlar veil (KV) physically stacked by fibers) were used to toughen composites as interleaves. Result shows that the existence of fusion-bonded dots strongly influenced the crack propagation and changed the interlaminar fracture mechanism. The Mode I fracture path of the nylon veil interleaved composite (NVIC) could propagate in the plane where the dots were located, whereas the path of the Kevlar veil interleaved composite (KVIC) randomly deflected inside the interlayer without the pre-cracking of the dots. The improvement of Mode I toughness was mainly based on fiber bridging and the resulting fiber breakage and pull-out. Fiber breakage was often observed for NVIC, whereas fiber pull-out was the main mechanism for KVIC. For the Mode II fracture path, the fusion-bonded NV dots guided the fracture path largely deflected inside the interlayer, causing the breakage of tough nylon fibers. The fracture path of the physically stacked KVIC occurred at one carbon ply/interlayer interface and only slightly deflected at fiber overlapped regions. Moreover, the fiber pull-out was often observed.

## 1. Introduction

The toughness of carbon fiber reinforced plastics (CFRPs) is strongly related to the interlaminar structure [1]. One of the most important methods for improving the impact resistance of CFRPs is interlayer toughening, which constructs the interlaminar structure and improves its toughness [2]. The interlayer toughening method introduces tough or strong materials into the interlayer of CFRPs through an interleaving technology. These tough or strong materials inside the interlayer play the roles of bridging, crack deflection, and viscoelastic energy dissipation, thereby significantly improving the energy dissipation density of crack propagation. Thus, the interlaminar toughness of CFRPs is effectively improved.

Numerous materials can be used as interleaves, such as non-woven veils [3], chopped fibers [4], thermoplastic particles or films [5], and nanoparticles [6,7]. Among these materials, non-woven veils formed by a thin layer of fibers (e.g., nylon veil [3], chopped Kevlar veil [4], and carbon fiber veil [8]) have attracted considerable attention as efficient materials for improving interlaminar fracture toughness. In recent years, veils formed by nano-fibers and nano-hybrid fibers with good toughening properties have gained considerable attention [9,10,11]. After being interleaved with veils, the Mode I interlaminar fracture toughness (*G*_IC_) and Mode II interlaminar fracture toughness (*G*_IIC_) of CFRPs are greatly improved. The improvement of *G*_IC_ and *G*_IIC_ is directly related to the improvement of compressive strength after impact [12,13].

Freestanding polymer veils are formed by polymer fibers through fusion bonding, adhesive bonding, physical stacking, or knitting. Polymer fibers are produced by a solution or by electrospinning, in which the diameter of solution-spun fibers is approximately 5–100 μm, and that of electro-spun fibers is tens of nanometers to several microns [14]. These veils have differences in fiber materials and veil structures, displaying different toughening properties for composites. Palazzetti, et al. (2014) studied the effects of veil thickness, fiber orientation, and diameter on the Mode I and Mode II fracture toughness [15]. Ramirez, et al. (2015) investigated the toughening properties of polyphenylene sulfide and polyether ether ketone veils. The effects of areal density, linearly density, and fiber diameter were discussed [16]. Heijden, et al. (2016) reported that polycaprolactone with different morphologies, such as nanofibers, microfibers, films, coatings, and microspheres, shows different toughening properties [17]. Kuwata, et al. (2011) studied veil materials, such as polyester, carbon fiber, polyamide (PA), and their hybrid, on interlaminar fracture toughness [18]. Saz-Orozco, et al. (2017) revealed the toughening properties of PA and polyethylene terephthalate (PET) veils. They found that the PET veil has better interfacial bonding but lower improvement of fracture toughness than the PA veil [19]. The increase in the interlaminar fracture toughness of interleaved composites is mainly due to the fiber bridging and the plastic zone ahead of the crack tip.

Crack propagation path is an important factor that affects the fracture mechanism and brings great changes in interlaminar fracture toughness. Hunt, et al. (2016) suggested that crack propagation presents various modes (i.e., stable propagation in the interlayer, stick-slip propagation, and stable propagation inside the carbon ply, with the condition of cure path, leading to the differences in *G*_IC_) [20]. Wang, et al. (2008) claimed that the crack propagation of composites at a high cooling rate is complex and causes extensive damage to the interlayer and breakage of the knit threads. *G*_IC_ is two times higher than the *G*_IC_ of the slow-cooled specimens [21]. In our previous work, we found that the aggregation of silver nanowires induces cracks to deflect into the high-toughness interlayer, leading to a significant increase in *G*_IIC_ [5]. However, firstly, although the fact that connection types exist objectively in the polymer veils, they were always skipped during research, including in applied studies [3] and theoretical simulations [22] of veil interleaved composites. Most studies are mainly focused on the effects of fiber structure, diameter, orientation, linearly density, and areal density on the interlaminar fracture toughness [3,4,8,15,16,17,18,19]. No research has reported on the effect of fiber connection types on the interlaminar fracture of composites. Secondly, most of these studies are only based on the fiber bridging mechanism of the veil interleaves [23], and studies on controlling the crack propagation path of veil interleaved composites are very rare [15,24]. In addition, finding a way to control the crack propagation path (or controlling the failure mechanism) is also very important for us to design the interleaf, not only for improving the mechanical properties of the composite, but also for new veil design for functionalized composite.

In this study, the effect of the fusion-bonded dots of veil interleaves on the crack propagation path of interleaved composites is investigated. Composites interleaved with another veil interleaf, which are physically stacked by fibers without any dots, are examined for comparison.

## 2. Experimental

### 2.1. Materials

The nylon veil was purchased from Beijing XinChengWei Import and Export Co., Ltd., Beijing, China. The Kevlar veil was provided by DuPont China Holding Co., Ltd., Beijing Branch, Beijing China. The apparent thickness of the veils was 53 μm and 55 μm, respectively, by testing the thickness of 10 layers of veils using a spiral micrometer. The areal densities were 16.3 g/m^2^ and 15.9 g/m^2^, respectively.

T800/5228 prepreg was a product of AVIC Composite Technology Center, Beijing, China. Here T800 was the reinforced carbon fiber (Areal density: ~130 g/m^2^). The 5228 resin was an aero-grade epoxy resin (Resin fraction of the prepreg: 34–35 wt%). The thickness of a single ply was 0.125 mm. Other auxiliary materials were purchased from commercial ways without further treatment.

### 2.2. Preparation of the Composite Laminates

The composite laminates for the interlaminar fracture toughness test were prepared using T800/5228 prepregs. First, the prepregs were stacked into preform. The stacking sequence was [0]_24_ according to China aviation industry standard HB7402-96 (for *G*_IC_ test) and HB7403-96 (for *G*_IIC_ test), where HB 7402-96 is based on the American Society for Testing and Materials (ASTM) standard D5528-01 [25], and HB 7402-96 is based on ASTM standard D790-00 [26]. Here [0]_24_ means that 24 carbon plies were stacked with the fibers orientation of 0° direction. One interleaf was inserted into the middle layer of the preform. A 25 μm thick polytetrafluoroethylene (PTFE) film was inserted into one end of the same middle layer at the region without the interleaf. The preform was finally cured in an autoclave according to the curing conditions of T800/5228 prepreg. The thickness of the laminates was controlled to 3.0 ± 0.1 mm.

The abbreviations of materials are given in Table 1:

### 2.3. Tests of Interlaminar Fracture Toughness

**Figure 1:**
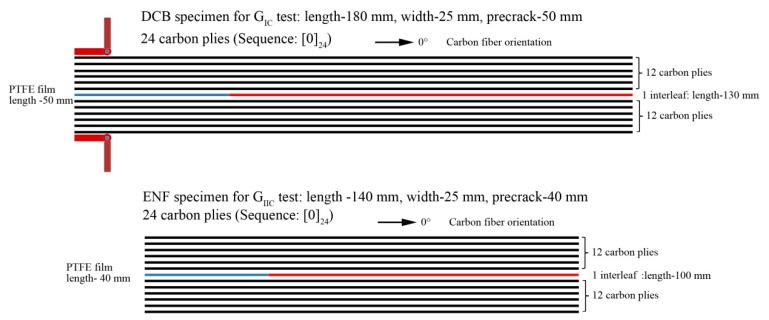
Schematic map of the geometries of the *G*_IC_ and *G*_IIC_ specimens.

The *G*_IC_ and *G*_IIC_ tests were according to HB7402-96 and HB740-96, respectively. The specimen geometries are illustrated in Figure 1. The *G*_IC_ test used a double-cantilever-beam method (DCB). The width and length of the *G*_IC_ specimens were 25 mm and 180 mm, respectively. The pre-crack made by PTFE was 50 mm long at one end of the specimen. The *G*_IIC_ test used end notched flexure specimens (ENF). The width and length of the *G*_IC_ specimen were 25 mm and 140 mm, respectively. The pre-crack made by PTFE film was 40 mm long at one end of the specimen. All the specimens was cut from the T800/5228 laminates with the stacking sequence of [0]_24_. Before testing, the specimens had an initial load applied to them to let the crack propagate 10 mm or 5 mm for *G*_IC_ and *G*_IIC_ tests, respectively. The calculation of *G*_IC_ and *G*_IIC_ can be used the equation given in [25,26].

### 2.4. Other Characterizations

Scanning electron microscopy (SEM) images were obtained by a Hitachi S-4800, Tokyo, Japan. All the samples were gold sprayed before testing. The optical micrographs were obtained by an Olympus SZ61 optical microscope, Tokyo, Japan. The metallographic micrographs were obtained by Leica Wetzlar 541000, Wetzlar, Germany.

## 3. Results and Discussion

### 3.1. Structure of Veils

**Figure 2:**
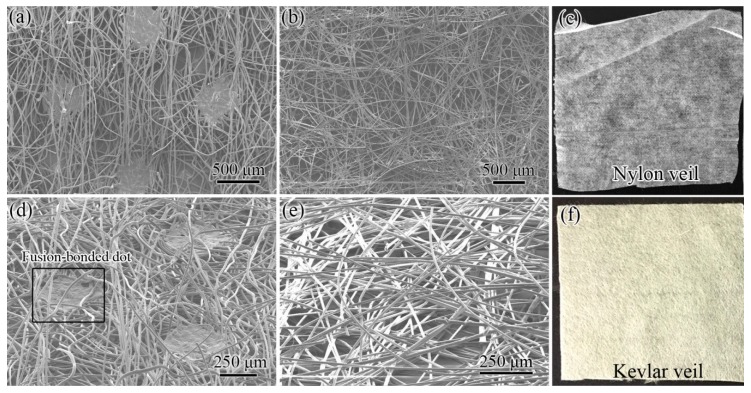
SEM images of the interleaves: (**a**) NV, (**b**) KV, (**d**) NV, 45° tilted, (**e**) KV, 45° tilted, and optical images of the (**c**) NV and (**f**) KV.

Two veils with different fiber connection types were used as interleaves, as shown in Figure 2. NV is a white veil formed by nylon fibers; its apparent thickness and areal density are 53 μm and 16.3 g/m^2^, respectively. KV is yellow because of the strong conjugation of benzene rings and amide groups. The apparent thickness and areal density of KV are 55 μm and 15.9 g/m^2^, respectively. Thus, the two veils have a similar thickness and areal density.

SEM images of the two veils have different structural geometries. NV is formed by several randomly distributed nylon fibers with an average diameter of 13 μm. The nylon fibers are connected by fusion-bonded dots (Figure 2a), which form a tetragonal lattice structure with a spacing of 1.25 mm. The diameter of the dots and number of dot per unit area are approximately 0.5 mm and 6.4 × 10^5^/m^2^, respectively. The dots connect the fibers, thereby forming an interconnected structure; thus, NV has tensile strength and nylon fibers cannot be pulled out from the veil. The 45° tilted SEM image of NV in Figure 2d shows that the nylon fibers are loose and evidently bended near the dots, indicating that the existing nylon dots induce a vertical alignment of nylon fibers. The thickness of the dots is smaller than the apparent veil thickness.

Different from NV, KV is physically stacked by numerous chopped Kevlar fibers without any bonding points; thus, KV has low tensile strength and Kevlar fibers can be easily pulled out from the veil (Figure 2b). The average length and diameter of the Kevlar fibers are 6 mm and 11 μm, respectively. In comparison with NV, the fiber distribution of KV is parallel to the veil plane without the restraint of fusion-bonded dots, as shown in Figure 2e.

Table 2 summarizes the structural characteristics of veil interleaves. The two veils have relatively similar areal densities, thickness, and fiber direction. The veil materials are all tough. Their main difference is that NV has numerous fusion-bonded dots for connecting fibers, whereas KV is only physically stacked by chopped fibers.

### 3.2. Interlaminar Fracture Toughness of Composites

**Figure 3:**
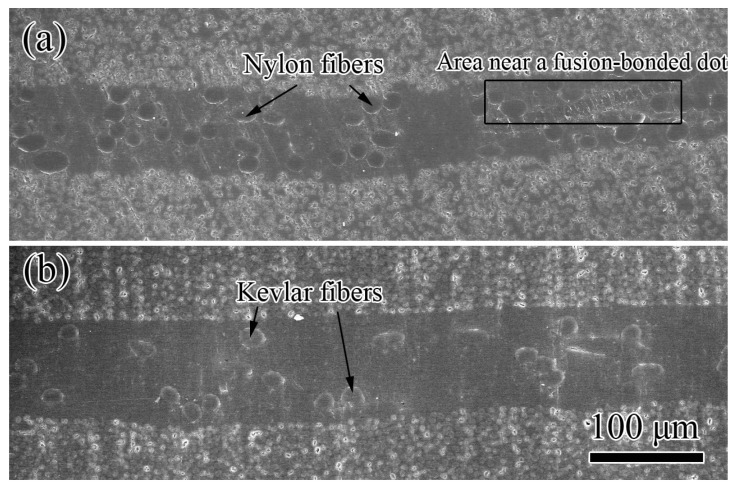
Cross sectional SEM images of the interleaved composites. (**a**) NVIC, (**b**) KVIC.

The epoxy matrix fulfilled the whole vision of the cross sectional SEM images of the interleaved composites, as shown in Figure 3, indicating there are no visible pores in the sample. Numerous brighter circles on the top and bottom parts can be identified as the cross section of carbon fibers because of their higher conductivity [27]. As the contrast, the interlayer containing non-conductive epoxy matrix and nylon/Kevlar fibers is much darker. Some elliptical circles with the sizes similar to the diameters of the nylon/Kevlar fibers are found, which can be considered as the cross section of nylon/Kevlar fibers. Thus the carbon plies and interlayers formed a laminated structure. Interleaves increase the interlayer thickness of composites. The interlayer thickness values of NVIC and KVIC obtained from the cross-sectional SEM images of interleaved composites are 67 and 74 μm, respectively. The small thickness of NV dots does not affect the interlayer thickness because of the bending and overlapping of nylon fibers, as shown in Figure 3a.

Table 3 presents the *G*_IC_ and *G*_IIC_ of the two interleaved composites and the control T800/5228 composite without any interleaf (Stacking sequence: [0]_24_; Thickness: 3.20 mm). The *G*_IC_ and *G*_IIC_ improvements of the NVIC are 118% and 236%, respectively, which are higher than those of the KVIC (72% and 171%, respectively). However, the interlaminar fracture toughness of CFRPs is related to numerous factors; thus, we only use it as reference values in this study.

### 3.3. Mode I Interlaminar Fracture

**Figure 4:**
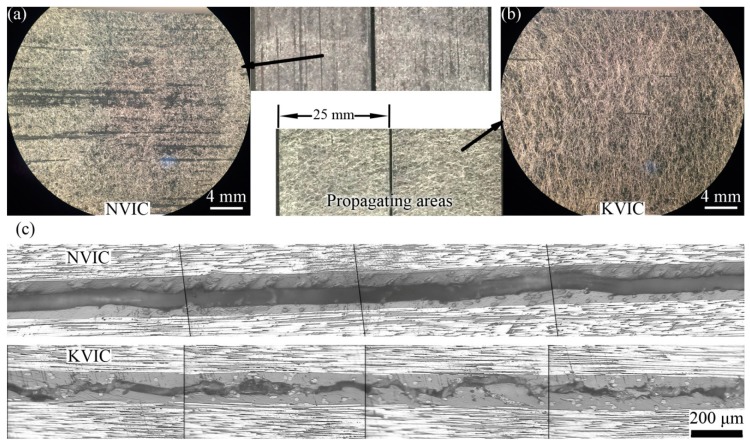
Optical micrographs and images of the Mode I fracture surfaces of the (**a**) NVIC and (**b**) KVIC, and (**c**) cross sectional metallographic micrographs of the Mode I fractured NVIC and KVIC samples.

The two fracture surfaces of NVIC or KVIC samples after the *G*_IC_ test have similar morphologies. Both surfaces are fully distributed with fibrous morphologies from the optical images and optical micrographs, as shown in Figure 4, indicating that the Mode I fracture occurs inside the interlayers containing interleaves. The diameters of veil fibers are smaller than the interlayer thickness, and fibers are distributed in multiple layers in the veils. Thus, several fibers are allotted to both sides of the fracture surfaces (Figure 4a,b). However, it can be seen from Figure 4c that there is a significant difference between the two crack paths. The crack path of NVIC is relatively smooth, mostly located in the middle layer of the interlayer, whereas the crack path of KVIC randomly spreads in the interlayer. The crack path of KVIC is also tortuous, and marks of fiber pull-out and resin fragments are often observed.

**Figure 5:**
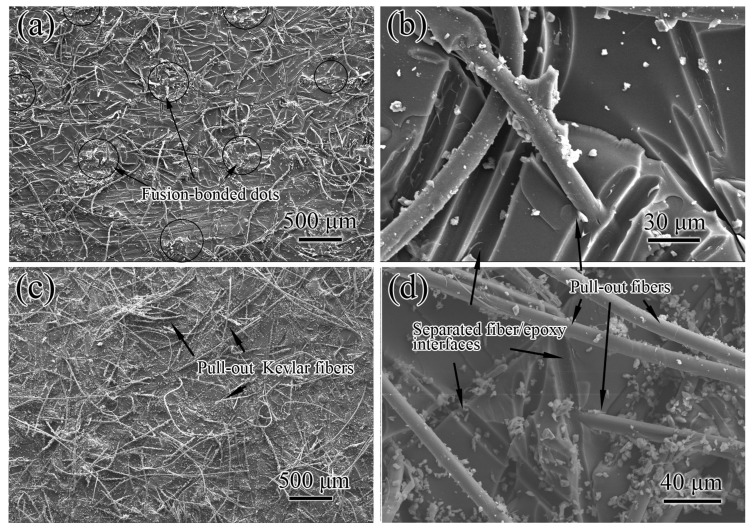
SEM images of the Mode I fracture surfaces of interleaved composites. (**a**,**b**) are NVIC, and (**c**,**d**) are KVIC.

The SEM observation provides a similar conclusion. Areas with the nylon dot like shapes can be seen in Figure 5a. They form square arrays and their surface topography is also similar to the nylon dots of the nylon veil. This indicates that the cracks happened at the nylon dot/epoxy matrix interfaces where the nylon dots are located. That is to say the Mode I fracture surface of NVIC occurs at the plane where the fusion-bonded dots are distributed inside the interlayer. The weak interfacial bonding of nylon and resin and the parallel distribution of dot surfaces may have caused dots to act as pre-cracks and guide the fracture path. Thus, the fracture path, which originally appears in the interlayer, occurs at the plane with the dots. Fiber bridging and breakage with residual epoxy resin appear everywhere on the fracture surface (Figure 5a,b). In addition, the improvement of *G*_IC_ can be attributed to fiber bridging and failure. The dots guide the crack propagation.

Numerous fiber pull-outs are observed from the Mode I fracture surface of KVIC (Figure 5c). The length of pull-out Kevlar is larger than that of the nylon fibers. Kevlar fibers are only physically stacked without the restriction of bonding dots. Fiber distribution is parallel to a ply plane, which is illustrated in Figure 2e. The main fracture mechanism of KVIC is fiber bridging and pull-out. Without restraint and crack guiding of fusion-bonded dots, the crack path of KVIC is more twisty than that of NVIC because of the random pull-out of Kevlar fibers and the corresponding breakage of the resin in the interlayer (Figure 5).

### 3.4. Mode II Interlaminar Fracture

**Figure 6:**
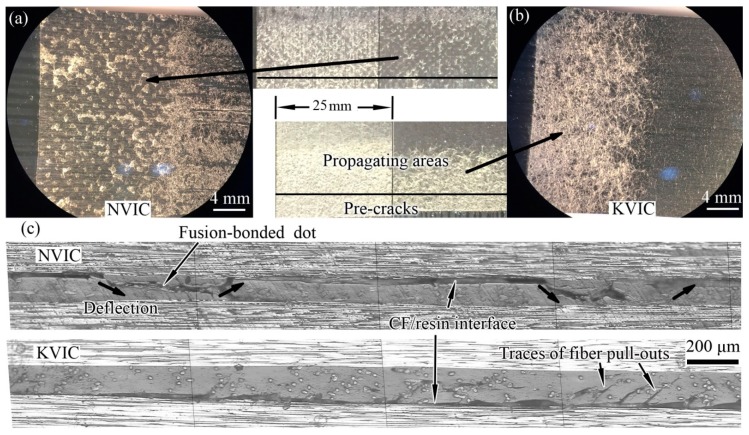
Optical micrographs and images of the Mode II fracture surfaces of the (**a**) NVIC and (**b**) KVIC, and (**c**) cross sectional metallographic micrographs of the Mode I fractured NVIC and KVIC samples.

The Mode II fracture surfaces of NVIC and KVIC samples have different morphologies, as displayed in Figure 6. Residual dot-like remnants are distributed on the fracture surface, thereby forming a tetragonal lattice structure. These dot-like remnants can be considered remnants of the interlayer at the regions of the nylon dots after Mode II fracture. For the KVIC sample, the Mode II fracture surface occurs at the carbon fiber/resin interface. At the initial region of the crack path, considerable fiber residues are found. These residues lessen along the crack propagation direction.

The crack paths presented in Figure 6c clearly support our discussion. The crack path of the NVIC sample mainly occurs at the interface of carbon ply and interlayer. However, their fracture zones are not all the same. It is clearly seen that the crack path deflects at specific regions, causing the damage of the interlayer in these areas. Striped distribution material, which is different from the epoxy resin, is observed at the deflection regions of the NVIC crack path. The length of these regions is approximately 0.5 mm, and the distance between the starting points of the neighboring deflection is approximately 1.74 mm, which agrees with the characteristics of the nylon fusion-bonded dots, as illustrated in Figure 2. For KVIC, the crack path occurs at the carbon ply/interlayer interface, with only a small deflection near the interface. Small cracks in the interlayer can be seen, which may be caused by the pull-out of Kevlar fibers.

**Figure 7:**
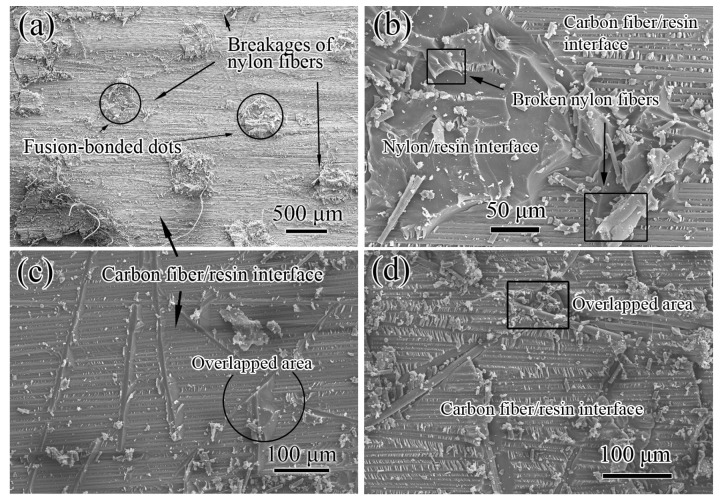
SEM images of the Mode II fracture surfaces of interleaved composites. (**a**,**b**) are NVIC, and (**c**,**d**) are KVIC.

The SEM image of the Mode II fracture surface also shows the tetragonal lattice distributed residues (Figure 7a). The crack occurs at the carbon fiber/resin interface and deflects at the location of the nylon dot, coinciding with Figure 6. From the enlarged image of one residue shown in Figure 7b, its debonding occurs at the nylon/resin interface similar to the Mode I fracture surface because of the poor interfacial bonding of nylon and resin, the planar surface, and their parallel distribution to the interlayer of the dots. Thus, the nylon fusion-bonded dots act as pre-cracks in the interlayer. These dots also evidently deflect the fracture path from the carbon fiber/resin interface to the nylon/resin interface inside the interlayer, causing the breakage of nylon fibers near the dots and achieving the great improvement of *G*_IIC_.

The Mode II fracture surface mainly occurs at the carbon fiber/resin interface for the KVIC sample, as displayed in Figure 7c,d, coinciding with Figure 6. Although the interfacial bonding of Kevlar and resin was also poor, the KVIC has the quite different crack propagation. Unlike the nylon dots, the Kevlar fiber/EP interface is circular and inconsistent with the direction of crack propagation; thus, the fibers rarely act as the pre-cracks inside the interlayer. Only for some fibers parallel to the crack direction and near the carbon ply/Kevlar fiber interface is the separation of the fiber/resin interface seen. The interface of Kevlar fiber pull-out with residual epoxy resin can be observed. The pull-out mostly occurs where the Kevlar fibers overlap. Thus, physically stacking fibers induces minor crack deflections. Different from the large and controllable crack deflections induced by fusion-bonded dots, the deflections were minimal and uncontrollable and occasionally caused the breakage of Kevlar fibers.

### 3.5. Discussion

**Figure 8:**
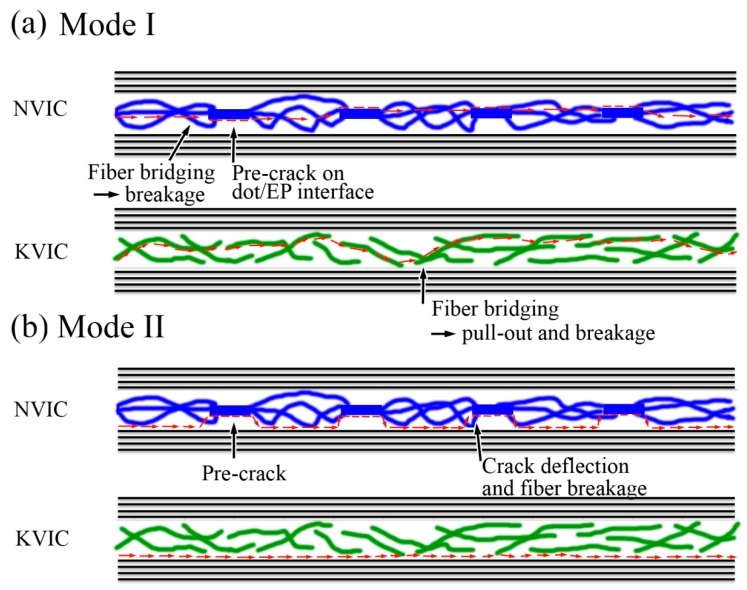
Sketch diagram of (**a**) Mode I and (**b**) Mode II fracture paths of the interleaved composites.

Figure 8 illustrated the crack propagation paths for the two veil interleaved composite. In the Mode I fracture, when the crack propagates in the interlayer containing sparse fibers, it also propagates in the interlayer [3,15,16,17,18]. The reason is that the low average interlayer toughness and the poor interface between fibers and resins cannot produce a plastic zone with a diameter larger than the interlayer thickness ahead of the crack tip [28]. For NV interleaves, fibers cannot be freely pulled out because of the restriction of fusion-bonded dots. In addition, dots act as pre-cracks when a crack propagates near them, thereby guiding the crack path to be deflected to the dot plane again. Thus, crack propagation mainly occurs in the plane where the bonding dots are located without large fluctuation (Figure 4c and Figure 8a). For KV interleaves formed by physically stacked Kevlar fibers, fibers can be easily pulled out without any restriction. The interlayer damage caused by disordered fiber pull-out renders the crack propagation unstable. Thus, the crack path is relatively tortuous (Figure 4c and Figure 8b).

In Mode II, the fracture often occurs at the carbon ply/interlayer interface with maximum shear stress for a composite interleaved with a tough material [3,5]. For NV interleaves, the fusion-bonded dots have a small thickness and act as pre-cracks. When propagating, the crack deflects from the ply/interlayer interface to the poor nylon/epoxy resin interface. The fusion-bonded dots also restrict the pull-out of fibers. Secondly, the nylon fibers are loose and evidently bended near the dots, and some are on the dot surface, as shown in Figure 2d, increasing the vertical alignment of nylon fibers. These two reasons cause the breakage of several nylon fibers near the dots during deflection (Figure 8b). For KV interleaves, the crack path only slightly deflects at the regions where fibers overlap near the ply/interlayer without the fusion-bonded dots acting as pre-cracks. The deflection process causes the pull-out of a certain amount of Kevlar fibers without the restriction of dots (Figure 8b).

Therefore, the fusion-bonded dots have two main effects. First, the dots produce large-scale poor interfaces between the tough interleaves and brittle resins inside the interlayer, which plays the role of pre-cracks, thereby guiding the crack propagation and causing crack deflection. Crack deflection further leads to a structural damage of the interlayer along the crack path, resulting in further energy dissipation. Second, the fusion-bonded dots restrict the free fiber pull-out and improve its vertical alignment, resulting in the increment of fiber bridging and the following breakage. Hence, we can control the fusion-bonded dots (or fiber connecting types) and the fiber geometries of veil interleaves to produce composites with high interlaminar fracture toughness as a further assumption.

## 4. Conclusions

A new way for controlling the crack propagation of veil interleaved composite by fusion-bonded dots of the veil was found. The fusion-bonded dots acted as pre-cracks inside the interlayer and restricted the fiber of the interleaves; thus, they guided the crack propagation and changed the interlaminar failure mechanism. For the Mode I fracture, the dots guided the crack propagation occurring at the plane where the dots were located. By contrast, the crack path of KVIC physically stacked by fibers randomly propagated inside the interlayer. For the Mode II interlaminar fracture, the pre-crack effect of the dots led the crack to regularly deflect from the carbon ply/interlayer interface to the nylon dot/resin interface, resulting in fiber breakage near the dots. By contrast, the crack path of KVIC physically stacked by fibers mainly propagated at one ply/interlayer interface and only slightly deflected at the overlapping area of Kevlar fibers. This crack propagation controlling method is will hopefully provide us with a new way to design the interleaf with a high toughening property.

## Figures and Tables

**Table 1:** Abbreviations of the interleaves and composites.

Interleaf	Abbreviation	Composite	Abbreviation
Nylon veil	NV	Nylon veil interleaved composite	NVIC
Kevlar veil	KV	Kevlar veil interleaved composite	KVIC

**Table 2:** Structural characteristics of the two veil interleaves.

Interleaf	Areal Density/(g·m^-2^)	Average Thickness/(μm)	Material	Diameter of Fibers/(μm)	Connection Type
NV	16.3	~53	Nylon	~13	Fusion-bonded dots
KV	15.9	~55	Kevlar	~11	Physically stacking

**Table 3:** The interlaminar toughness of three interleaved composites.

Sample	Control [3]	NVIC	KVIC
*G*_IC_/(J/m^2^)	306	666	526
Std. Dev./(J/m^2^)		37.2	22.6
*G*_IIC_/(J/m^2^)	718	2410	1946
Std. Dev./(J/m^2^)		95	57
